# Epidemiological Characterization of Influenza A(H1N1)pdm09 Cases from 2009 to 2010 in Baguio City, the Philippines

**DOI:** 10.1371/journal.pone.0079916

**Published:** 2013-11-11

**Authors:** Rochelle R. Pamaran, Taro Kamigaki, Teresita T. Hewe, Korrine Madeleine C. Flores, Edelwisa S. Mercado, Portia P. Alday, Alvin G. Tan, Hitoshi Oshitani, Remigio M. Olveda, Veronica L. Tallo

**Affiliations:** 1 Research Institute of Tropical Medicine, Manila, Philippines; 2 Department of Virology, Tohoku University Graduate School of Medicine, Sendai, Japan; Arizona State University, United States of America

## Abstract

**Background:**

Baguio City, Philippines experienced its first influenza A(H1N1)pdm09 [A(H1)pdm09] case in May 2009. In spite of numerous reports describing the epidemiological and clinical features of A(H1)pdm09 cases, there are no studies about A(H1)pdm09 epidemiology in the Philippines, where year-round influenza activity was observed.

**Objectives:**

We aimed to investigate the epidemiological and clinical features of A(H1)pdm09 in pandemic and post-pandemic periods.

**Methods:**

Data were collected under enhanced surveillance of influenza-like illness (ILI) and severe acute respiratory infection (SARI) from January 2009 to December 2010. RT-PCR was used to detect A(H1)pdm09, following the protocol of the United States Centers for Disease Control and Prevention. The reproduction number was computed as a simple exponential growth rate. Differences in proportional and categorical data were examined using chi-square test or Fishers’ exact test.

**Results and Conclusions:**

The outbreak was observed from week 25 to 35 in 2009 and from week 24 to 37 in 2010. The highest proportion of cases was among children aged 5–14 years. The number of ILI outpatients was 2.3-fold higher in 2009 than in 2010, while the number of inpatients was 1.8-fold higher in 2009. No significant difference in gender was observed during the two periods. The clinical condition of all patients was generally mild and self-limiting, with only 2 mortalities among inpatients in 2009. The basic reproduction number was estimated as 1.16 in 2009 and 1.05 in 2010 in the assumption of mean generation time as 2.6 days. School children played a significant role in facilitating influenza transmission.

## Introduction

Baguio City is an urbanized city located in the highland of northern Luzon, Philippines with over 298,500 inhabitants. It has a population density of 5,190 persons/km^2^. Children aged <5 years and teenagers comprised 10.1% and 22.2%, respectively, of the total population, while the elderly (age, >60 years) comprised only 5.0% [1]. Thus, the population structure is largely clustered in the working age group.

In early April 2009, the first human case of influenza A(H1N1)pdm09 [A(H1)pdm09] was identified in Mexico and the United States [2]. This virus then spread rapidly to other countries. The Philippines identified its earliest case on May 22, 2009, and Baguio City reported its first A(H1)pdm09 cases 3 days later. The timing of the initial introduction in mid-May was similar to that of other neighboring Asian countries [3-8]. A total of 159 cases and 8 fatalities was officially reported in the Cordillera Administrative Region, where Baguio City is located, by 31 August, 2009 [9].

Prior to the emergence and spread of A(H1)pdm09, enhanced surveillance of influenza-like illness (ILI) and severe acute respiratory infection (SARI) was conducted as part of the Influenza Disease Burden study (BOD study) in the Baguio City. The study aimed to estimate influenza disease burden through surveillance in 16 city district health centers and 5 hospitals in the city. In brief, this system detected overall 508 outpatients and 98 inpatients who were positive for A(H1)pdm09 from May 2009 to Dec 2010.Numerous reports have described the epidemiological and clinical features of A(H1)pdm09 cases; however, they mostly covered the pandemic periods and few covered the post-pandemic period [10]. Moreover, there was no study about A(H1)pdm09 epidemiology in the Philippines, where year-round influenza activity was observed. This present study therefore aimed to describe the epidemiological and clinical features of A(H1)pdm09 both during the pandemic and post-pandemic periods.

## Materials and Methods

### Study Period and Population

Data were collected through ILI and SARI surveillance from January 2009 to December 2010. In brief, ILI was defined as a case with fever and cough and/or sore throat (or runny nose in children aged ≤3 years), and SARI was defined as a case having all ILI symptoms and difficulty in breathing, requiring hospital admission. Data and specimens were collected by influenza surveillance nurses. These data obtained from enhanced surveillance, conducted as part of the BOD study, were studied to estimate both outpatient and inpatient incidence rates (unpublished data). Epidemiological data obtained from cases on a standardized case report form included age, gender, nationality, comorbidities, date of onset of symptoms, history of illness, physical examination findings, and vaccination status of patients. Naso- and/or oropharyngeal swabs were obtained from the identified cases. Specimens were stored in a refrigerator and then shipped to the Research Institute of Tropical Medicine (RITM). RT-PCR testing was used to detect A(H1)pdm09 at the Molecular Biology Laboratory in RITM using a protocol of the United States Centers for Disease Control and Prevention [11]. A trained surveillance nurse explained the purpose of this study and obtained both verbal and written consent to participate in this surveillance study either directly from cases or from parents or guardians if a case was children or minors. After obtaining participants’ consent, the nurse started filling the case report form and collecting the sample. Those forms were securely archived in the office. The scheme of this surveillance study was reviewed and approved by the RITM Institutional Review Board on March 31, 2009.

### Reproduction Number Estimation

The basic reproduction number (R0) is defined as the expected number of secondary cases generated by a primary case in a completely susceptible population. This parameter was widely used as a part of expression of transmissibility in the community. Among several possible methods to estimate R0, we used a simple exponential growth rate approach that has been documented elsewhere [12-14]. The exponential growth rate (r) was estimated by fitting the daily number of cases, which occurred in the exponential phase of waves in 2009. The reproduction number was then estimated by substituting r into the following formula; 

R0=(1+r/b1)×(1+r/b2) (1)

where 1/b1 and 1/b^2^ are the mean latent period and infectious period, respectively. Thus, the mean generation time is given as 1/b1+1/b2. We used two mean generation times 2.6 days and 4 days, as estimated in other studies [15,16].

### Statistical Analyses

We used the 2007 census population as the standardized population to estimate both outpatient and inpatient incidences in this study. Using the occupation data for cases, we constructed the weekly ratio of students and non-students with A(H1)pdm09 during the growth phase of the epidemic (week 21-week 39, 2009). The ratio was then calculated by dividing the number of students by the number of non-students over those weeks. Any differences in the proportional and categorical data were examined using Fisher’s exact test or chi square test. All data were compiled using Microsoft excel (Microsoft, WA) and analyzed using PASW 20.0 (IBM, NY).

## Results

The number of A(H1)pdm09 outpatients was 2.3-fold higher in 2009 than in 2010, and the number of inpatients was 1.8-fold higher in 2009 (Table 1). For the 2-year period, the outpatient incidence was highest among children aged <5 years, followed by the individuals aged 5–14 years, and lowest among those aged ≥50 years. The distribution by sex and age group was similar between outpatients and inpatients for both periods, except among those aged ≥50 years, who accounted for 11.5% among inpatients and 1.1% in outpatients. Among cases with the data about their occupations (22%), students were most frequently recorded. Elementary school students accounted for 66% and 71% of all students in 2009 and 2010, respectively. Housewives and unemployed individuals were significantly higher among both outpatients and inpatients in 2009 than in 2010 (*p* < 0.001) (Table 2).

**Table 1:** Demographic characteristic of outpatients and inpatients with influenza A(H1N1)pdm09 in Baguio City for 2009 and 2010.

Category		**Outpatients**							**Inpatients**							
		**2009** (N = 356)			**2010** (N = 152)			*p* value	**2009** (N = 69)			**2010** (N = 29)			*p* value	
		No.	%	rate *	No.	%	rate*		No.	%	rate*	No.	%	rate*		
**Overall**				11.9			5.1				1.8			0.9		
**Age**	<5	137	38.5	45.5	62	40.8	20.6	0.19	26	37.7	8.6	12	41.4	3.9		0.63
	5–14	155	43.5	27.9	72	47.4	12.9		19	27.5	3.4	4	13.8	0.7		
	15–29	48	13.5	4.5	11	7.2	1		7	10.1	0.7	3	10.3	0.3		
	30–49	12	3.4	1.7	7	4.6	0.9		9	13.0	1.2	5	17.2	0.7		
	≥50	4	1.1	1.2	0	0.0	0		8	11.6	2.3	5	17.2	1.5		
**Sex**	Male	183	51.4		75	49.3		0.70	36	52.2		20	69.0			0.18
	Female	173	48.6		77	50.7			33	47.8		9	31.0			

* rare per 10,000 population

**Table 2:** Occupational information and Vaccine status among outpatients and inpatients with influenza A(H1N1)pdm09 in Baguio City for 2009 and 2010.

Category		**Outpatients**					**Inpatients**				
		**2009**		**2010**		*p* value	**2009**		**2010**		*p* value
		No.	%	No.	%		No.	%	No.	%	
**Occupations**	Students	189	53.1	75	49.3	0.10	21	30.4	7	24.1	0.22
	Health workers	2	0.6	1	0.7	1	1	1.4	0	0	n/a
	Other workers	19	5.3	7	4.6	0.56	10	14.5	7	24.1	0.06
	Housewives	9	2.5	1	0.7	<0.001	1		0	0	n/a
	Inoccupation	49	13.8	1	0.7	<0.001	1	1.45	15	51.7	<0.001
**Seasonal flu Vaccination**		3	0.8	4	2.6	0.03	4	5.8	3	10.3	0.21

A(H1)pdm09 outpatients generally had ILI signs and symptoms without any symptoms and comorbidities; a few had diarrhea, vomiting, abdominal pain, loss of appetite and 2 cases reported pregnancy (Table 3). Clinical manifestation and physical examination findings among confirmed inpatients between 2009 and 2010 were similar. Almost half of the outpatients (49%) were prescribed antibiotics, while only 1 was prescribed antivirals. There observed no difference of median duration of illness prior to consultation between 2009 and 2010 (Table 4).

**Table 3:** Summary of clinical symptoms, physical examinations, and underlying conditions of cases with influenza A(H1N1)pdm09 in Baguio City for 2009 and 2010.

**Category**	**Subcategory**	**Outpatients**					**Inpatients**				
		**2009**		**2010**		*p* value	**2009**		**2010**		*p* value
		No.	%	No.	%		No.	%	No.	%	
Symptoms	Fever	356	100	152	100		64	92.8	27	93.1	0.91
	Cough	339	95.2	144	94.7	0.68	66	95.7	28	96.6	0.68
	Runny nose	313	87.9	135	88.8	0.59	50	72.5	23	79.3	0.16
	Sore throat	129	36.2	46	30.3	0.01	13	18.8	9	31.0	0.03
	DoB	63	17.7	18	11.8	<0.001	44	63.8	21	72.4	0.11
	Headache	203	57.0	74	48.7	0.001	22	31.9	11	37.9	0.30
	Muscle ache	85	23.9	50	32.9	<0.001	21	30.4	7	24.1	0.22
	Vomiting	34	9.6	10	6.6	0.02	17	24.6	11	37.9	0.02
	Loss of appetite	34	9.6	23	15.1	0.003	9	13.0	2	6.9	0.50
	Diarrhea	6	1.7	3	2.0	0.69	6	8.7	4	13.8	0.48
	Abdominal pain	12	3.4	4	2.6	0.38	2	2.9	0	0.0	n/a
Physical examinations	Fever (>38.0)	103	28.9	36	23.7	0.02	35	50.7	11	37.9	0.03
	Wheezing	n/a		n/a			16	23.2	5	17.2	0.78
	Chest indrawing	2	0.6	0	0.0	n/a	14	20.3	4	13.8	0.11
	Abnormal findings in X-ray	n/a		n/a			34	49.3	12	41.4	0.18
Underlying medical condition	Asthma	1	0.3	n/a		n/a	21	30.4	9	31.0	0.91
	DM	n/a		n/a			4	5.8	0	0.0	0.31
	CVD	n/a		n/a			5	7.2	3	10.3	0.69
	COPD	n/a		n/a			2	2.9	4	13.8	0.07
	Pregnancy	2	0.6	0	0.0	n/a	0	0.0	0	0.0	n/a

**Table 4:** Summary of clinical course and management of cases with influenza A(H1N1)pdm09 in Baguio City for 2009 and 2010.

**Category**	**Subcategory**	**Outpatients**					**Inpatients**				
		**2009**		**2010**		*p* value	**2009**		**2010**		*p* value
		No.	%	No.	%		No.	%	No.	%	
Medical procedures	Antibiotics	176	49.4	71	46.7	0.30	61	88.4	27	93.1	0.12
	Antiviral	1	0.3	0	0	n/a	25	36.2	0	0.0	<0.001
	Oxygenation	n/a		n/a			23	33.3	7	24.1	0.07
	Steroids	0	0.0	0	0.0	n/a	8	11.6	3	10.3	0.73
	Intubation	n/a		n/a			5	7.2	0	0.0	0.32
Outcomes	Recovered	n/a		n/a			64	92.8	27	93.1	1
	Deaths	n/a		n/a			2	2.9	0	0.0	1
Median duration of illness prior to consultation		2 days (0–12 days)		2 days (1–5 days)			3 days 0–31 days)		4 days (1–15 days)		
Median duration of hospitalization		n/a		n/a			3 days (1–26 days)		3 days (1–38 days)		
Median duration of antivirals prescription		n/a		n/a			5 days (5–10 days)				
Median duration between onset and antiviral prescription		n/a		n/a			3 days (1–36 days)				

Cases were admitted within 3–4 days of onset of illness, with the majority presenting with signs of SARI, including difficulty in breathing, chest indrawing, and wheezing on physical examination. Asthma was the predominant comorbidity in almost a third of the cases for both periods, while a few had diabetes mellitus (DM) (5.8% vs. 0%), cardiovascular disease (CVD) (7.2% vs. 10.3%), and chronic obstructive pulmonary disease (COPD) (2.9% vs. 13.8%) as underlying conditions (Table 3). Abnormal radiological findings (49.3% vs. 41.4%) of pneumonia were also similar between the pandemic and post-pandemic years. The majority of cases during the two periods were treated with antibiotics (88.4% vs. 93.1%), while only 36.2% were treated with antivirals in 2009. There were 33.3% and 24.1% patients who received supplemental oxygen in 2009 and 2010, respectively, and 5 were intubated while in the wards in 2009. The majority, except for 2 fatalities in 2009, were discharged recovered after 3 days of confinement. The first fatality was of a 56-year-old male with several underlying conditions, such as COPD, hypertensive CVD, myocardial infarction, and DM, who was admitted 6 days after the onset of illness. He was treated with antivirals on the 2nd day of confinement and intubated, but he died after 7 days. The other fatality was of a 40-year-old male with DM and chronic renal disease who was admitted 6 days after the onset of illness. The patient was treated with antivirals after 6 days of confinement and intubated, but he died after 30 days of hospitalization.

**Figure 1:**
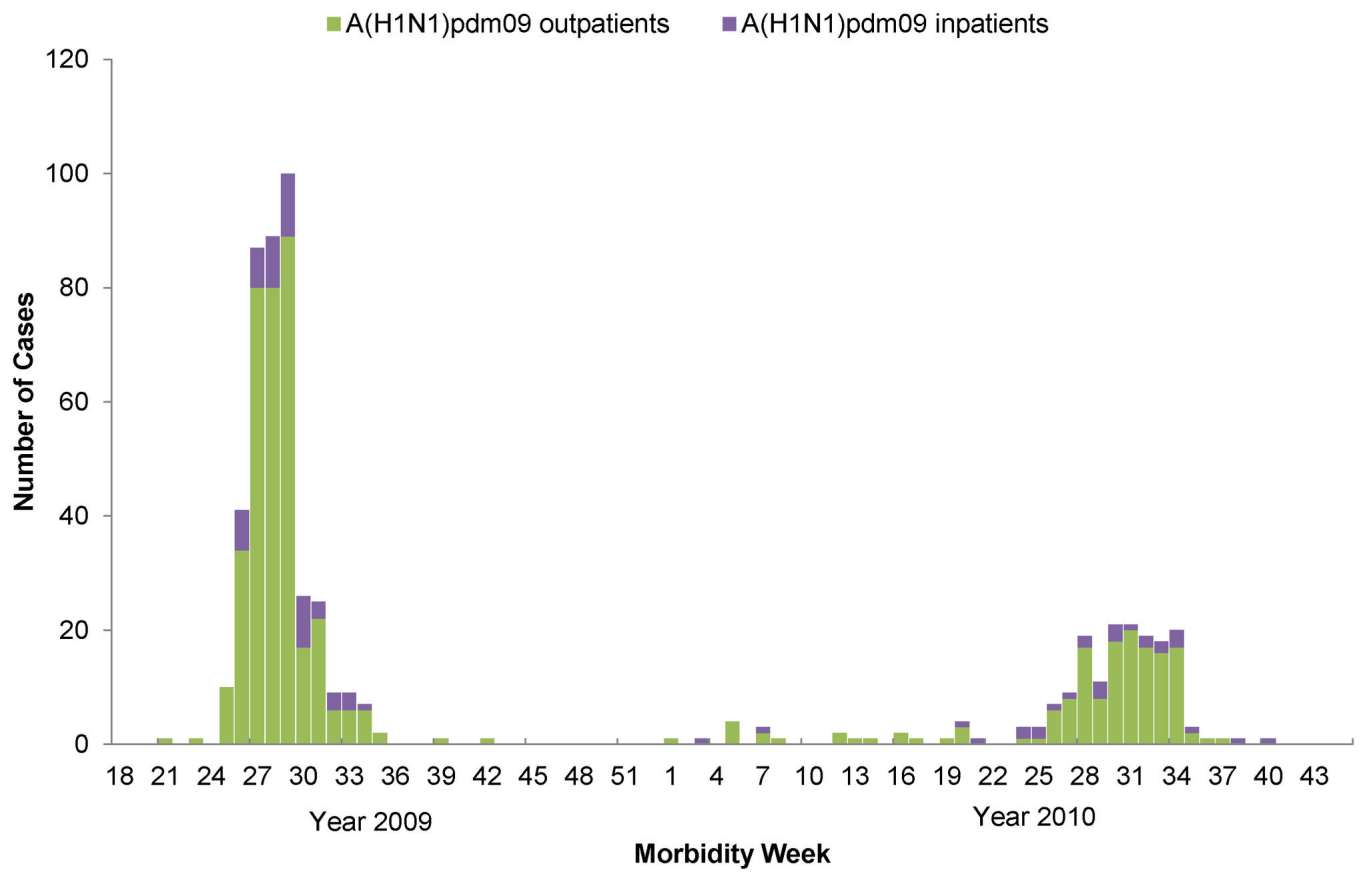
Weekly number of influenza A(H1N1)pdm09 cases in outpatients and inpatients, Baguio City, week 18, 2009 to week 45, 2010.

Figure 1 shows the weekly number of A(H1)pdm09 cases among outpatients and inpatients. The outbreaks were observed during morbidity weeks 25–35 in 2009 and weeks 24–37 in 2010. There were fewer confirmed cases sporadically distributed between these two epidemics. On the basis of the number of cases during the growth phase, we estimated the basic reproduction number of 1.14 (95% confidence interval (CI) 1.13–1.15) in 2009 (from June 18 to July 6) and 1.09 (95% CI 1.08–1.10) in 2010 (from June 18 to July 11), with a mean generation time (MGT) of 2.6 days (Table 5). These numbers were altered to 1.09 and 1.06 with MGT of 4 days in 2009 and 2010, respectively (Figure S1).

**Table 5:** The estimation of reproduction number from the growth phase of epidemics due to influenza A(H1N1)pdm09 in 2009 and 2010.

**Year**	**Mean generation time (2.6 days)**	**Mean generation time (4 days)**
2009	1.14(1.13–1.15)	1.09 (1.08–1.10)
2010	1.09 (1.08–1.10)	1.06 (1.05–1.07)

**Figure 2:**
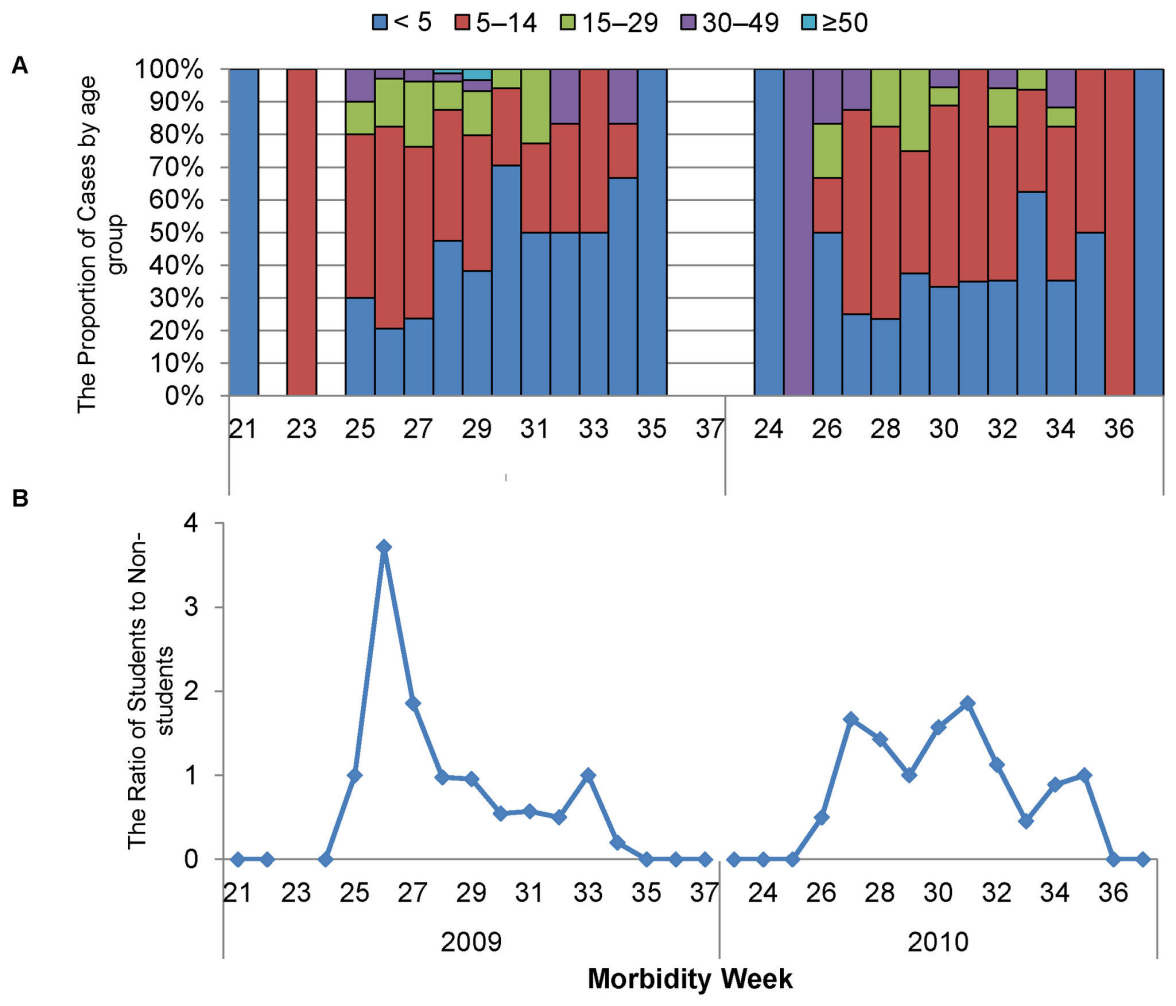
A-B. Weekly proportion of influenza A(H1N1)pdm09 cases by each age group (A) and weekly ratio of students to non-students, week 21, 2009 to week 37, 2010, Baguio City.

Figure 2A and B shows the weekly ratio of students to non-students together with the proportion of age groups during outbreaks of 2009–2010. We observed over 1-fold ratio in the growth phase of peaks in both outbreaks. The ratio increased a week prior to the peak of confirmed cases. This peak ratio was obviously higher in 2009 than in 2010 (3.7 and 1.9, respectively). After the growth phase, the ratio scaled down to near 1.0. The highest proportion of cases was observed among children aged <5 years.

## Discussion

In this study, we investigated influenza A(H1)pdm09 epidemiology from 2009 to 2010 in Baguio City. The total number of cases reported in 2009 was much higher than in 2010, and the highest number of cases was observed in the 5 to 14-year-old group, most of which were school-age children. This is consistent with findings from a study of 8 countries that estimated the highest cumulative incidences of A(H1)pdm09 among school-age children [17]. Ten of the 13 inpatients belonged to the >50-year-old group were aged <65 years. This younger age shift of inpatients could explain the lower mortality observed in this study. The ratio of deaths to hospitalizations due to A(H1)pdm09 was the highest among the >65-year-old group [18] partly because those who are aged >80 years have pre-existing immunity against A(H1)pdm09 [19,20].

There were two epidemics in June–August of each year, while cases were sparsely distributed in between (Figure 1).We did not observe a wave during the cooler months of September–November in 2009, which was widely observed in neighboring countries and cities[21-24] but not in Malaysia [25]. The increasing number of cases occurred mainly in June when schools in the country were opened. This coincidence between the resumption of classes and expanding transmission in the community has been described in other studies [14,26]. With a higher ratio of students to non-students, especially in the growth phase of epidemics, A(H1)pdm09 transmission was multiplied among students who play a role in expanding transmission of influenza in the community [27]. Moreover, housewives and unemployed individuals were significantly higher among both outpatients and inpatients in 2009 than in 2010. This may be because housewives are more likely to take care of patients at home. Household transmission could be one of the important opportunities for A(H1)pdm09 to spread, as reported in other studies [28,29], but its qualitative risk is difficult to measure. 

We estimated R0 as 1.14 in the growth phase of the 2009 wave. A review of transmission parameters for the (H1N1)2009 pandemic indicated the median reproduction number as 1.6 (1.1–3.1) [16], and a higher value was mostly obtained from outbreak settings in schools [30,31]. This variability could be explained as a reflection of differing estimation methods and assumptions regarding generation time and the under-reporting rate. Heterogeneity resulting from control measures undertaken as well as a wave period over the entire pandemic period could affect the estimation of reproduction number. A study that investigated the reproduction number in the southern hemisphere demonstrated a positive correlation between the proportion of children aged <20 years and reproduction number [32]. The fact that 45% of A(H1)pdm09 cases were children aged <20 years may have led to a potentially higher reproduction number in the city. The reasons why we had a lower basic reproduction number remain unclear. Baguio City is located in the highland and has a mean temperature of 19.6°C and mean relative humidity of 88.1%. This could have decreased the effective transmissibility of influenza virus [33]. Moreover, a social contact study in Vietnam revealed the concentration of close contact in home settings, although the physical contact levels were low [34]. This pattern of close contact in home settings would be similar in the Philippines. In this regard, additional study focusing on social contacts and the dynamics of influenza in the community is necessary.

The clinical condition of outpatients was generally mild and self-limiting. Some 34% of inpatients had developed wheezing or chest indrawing, and 12 of the 21 cases who developed wheezing did not have asthma as their comorbidity. A study in Japan indicated that about a third of the cases who had asthma attacks during A(H1)pdm09 infection were not diagnosed to have asthma [35]. A(H1)pdm09 infection may lead to an aberrant asthmatic response although this mechanism remains unclear. No fatal cases were reported although asthma was the most common comorbidity among inpatients. This observation was similar to other studies in that asthma is observed as one of the risk factors for hospitalization but is not directly correlated with fatal severity [18,35-37]. In terms of intensive clinical severity, 33% of inpatients were administered oxygen in total and 7.2% were intubated with 2 deaths in 2009. None was intubated and expired in 2010.

In the Philippines, antivirals are not commonly used for ILI treatment. Only the laboratory-confirmed cases were administered antivirals, subject to the time and condition of patients when the laboratory results arrived. During the (H1N1)2009 pandemic, interim guidelines on use of antivirals were issued by the national government, particularly the Department of Health, which encouraged physicians to prescribe antivirals. Therefore, 25 inpatients were administered Oseltamivir in 2009. We could not see a linear correlation between intervals from onset of medication and duration of hospitalization (*p* = 0.64). Another study demonstrated that early treatment with antivirals leads to better clinical outcomes [38], but this study did not show similar results. This is partly because 75% of cases were administered antivirals after admission, which suggests that cases already developed SARI conditions, and because of the small number of cases.

There were some limitations in this study. First, we detected influenza A(H1)pdm09 among outpatients at the city health centers and in one of outpatients’ department of a tertiary hospital. Thus, we may have potentially missed cases that did not seek consultation or consulted at other health facilities such as private clinics. Second, our reproduction estimation was truly based on the exponential growth rate and known generation times studied in other countries. In spite of this limitation, this method has been widely used [12-14]. Third, because the updated census (2010 population census) data were not available as age-stratified figures, we may be overestimating rates because of a lower population in the 2007 census.

In conclusion, the demographic and clinical characterization for A(H1N1)pdm09 cases shown in this study were similar to that of other countries. We observed epidemics due to A(H1)pdm09 in July–August of both years but not during the cooler months of September–December of 2009. School-age children played a relatively significant role in expanding transmission in the city, while lower a reproduction number was estimated in the growth phase. During the 2-year study period, only 2 fatalities were recorded. There was relatively quick medical attention for both outpatients and inpatients during the pandemic period although the frequency of antiviral prescription was significantly low. This reinforces the importance of the growing capability to provide appropriate medical consultation in the community. Finally, it is important to establish surveillance systems to monitor influenza activity, which will provide helpful information to both stakeholders and the public for implementing a pandemic influenza response.

## Supporting Information

Figure S1 **A-B. Cumulative number of daily reported influenza A(H1N1)pdm09 cases and fitted exponential curve in 2009 (A) and 2010 (B).**
(TIFF)
