# Supplementary figures and images for: Epidemiological Characterization of Influenza A(H1N1)pdm09 Cases from 2009 to 2010 in Baguio City, the Philippines
Source: PLoS One. 2013 Nov 11;8(11):e79916. doi: 10.1371/journal.pone.0079916 (PMC3823603; doi:10.1371/journal.pone.0079916)

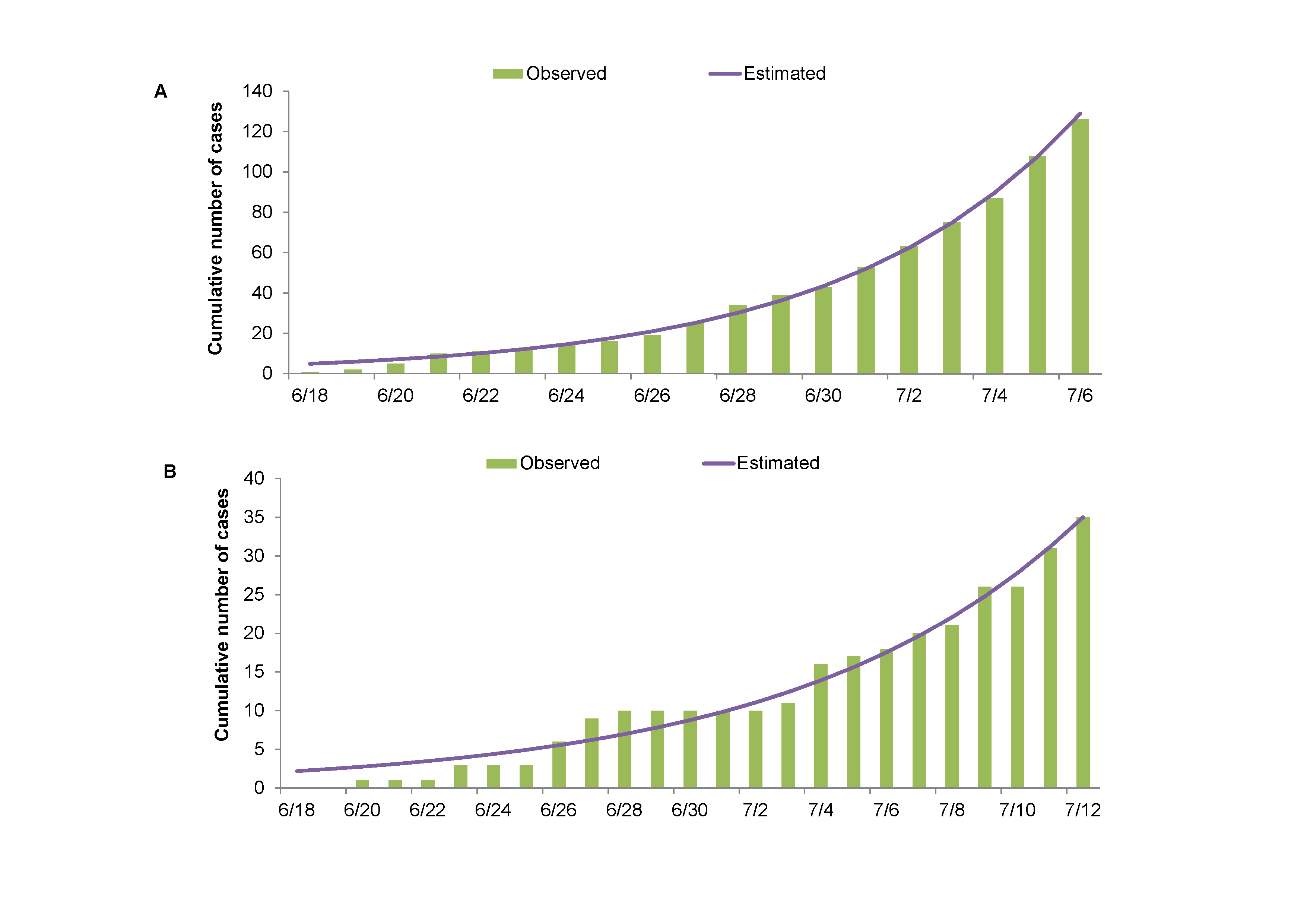

Supplement: Figure S1 — A-B. Cumulative number of daily reported influenza A(H1N1)pdm09 cases and fitted exponential curve in 2009 (A) and 2010 (B). (TIFF) [file pone.0079916.s001.tiff]
